# Recessive mutations in *ATP8A2* cause severe hypotonia, cognitive impairment, hyperkinetic movement disorders and progressive optic atrophy

**DOI:** 10.1186/s13023-018-0825-3

**Published:** 2018-05-31

**Authors:** Hugh J. McMillan, Aida Telegrafi, Amanda Singleton, Megan T. Cho, Daniel Lelli, Francis C. Lynn, Julie Griffin, Alexander Asamoah, Tuula Rinne, Corrie E. Erasmus, David A. Koolen, Charlotte A. Haaxma, Boris Keren, Diane Doummar, Cyril Mignot, Islay Thompson, Lea Velsher, Mohammadreza Dehghani, Mohammad Yahya Vahidi Mehrjardi, Reza Maroofian, Michel Tchan, Cas Simons, John Christodoulou, Elena Martín-Hernández, Maria J. Guillen Sacoto, Lindsay B. Henderson, Heather McLaughlin, Laurie L. Molday, Robert S. Molday, Grace Yoon

**Affiliations:** 1 Division of Neurology, Department of Pediatrics, Children’s Hospital of Eastern Ontario Research Institute, University of Ottawa, Ottawa, ON Canada; 2 GeneDx, Gaithersburg, MD USA; 3 Division of Neurology, Department of Medicine, The Ottawa Hospital, University of Ottawa, Ottawa, ON Canada; 4 Diabetes Research Program, Child and Family Research Institute, Vancouver, BC Canada; 5 Department of Surgery and Department of Cellular and Physiological Sciences, University of British Columbia, Vancouver, BC Canada; 6 Weisskopf Child Evaluation Center, Department of Pediatrics, School of Medicine, University of Louisville, Louisville, KY USA; 7 Department of Human Genetics, Donders Institute for Brain, Cognition and Behaviour, Radboud University Medical Center, Nijmegen, The Netherlands; 8 Department of Neurology, Donders Center of Neuroscience, Radboud University Medical Center, Nijmegen, The Netherlands; 9 Department of Neurology, Donders Institute for Brain, Cognition and Behaviour, Radboud University Medical Center, Nijmegen, The Netherlands; 10 Assistance Publique Hôpitaux de Paris, Département de Génétique, Groupe Hospitalier, Pitié-Salpêtrière, Paris, France; 11 Service de Neuropédiatrie, Hôpital Armand-Trousseau, Paris, France; 12 Centre de Référence Déficiences Intellectuelles de Causes Rares, GH Pitié Salpêtrière, Paris, France; 13 Groupe de Recherche Clinique UPMC Déficience Intellectuelle de Causes Rares et Autisme GH Pitié-Salpêtrière, Paris, France; 14 Genetics Program, North York General Hospital, Toronto, ON Canada; 15 Medical Genetics Research Centre, Shahid Sadoughi University of Medical Sciences, Yazd, Iran; 16 Reproductive Sciences Institute, Shahid Sadoughi University of Medical Sciences, Yazd, Iran; 17 Diabetes Research Centre, Shahid Sadoughi University of Medical Sciences, Yazd, Iran; 18 Human Genetics Research Centre, Molecular and Clinical Sciences Institute, St George’s University of London, London, UK; 19 Department of Genetic Medicine, Westmead Hospital, Westmead, NSW Australia; 20 Sydney Medical School, University of Sydney, Sydney, NSW Australia; 21 Institute for Molecular Bioscience, University of Queensland, St Lucia, QLD Australia; 22 Neurodevelopmental Genomics Research Group, Murdoch Childrens Research Institute and Department of Paediatrics, Melbourne Medical School, University of Melbourne, Melbourne, VIC Australia; 23 Unidad de Enfermedades Mitocondriales-Metabólicas Hereditarias, Servicio de Pediatría Hospital Universitario 12 de Octubre, Universidad Complutense de Madrid, Madrid, Spain; 24 Department of Biochemistry and Molecular Biology, University of British Columbia, Vancouver, BC Canada; 25 Department of Ophthalmology and Visual Sciences, Centre for Macular Research, University of British Columbia, Vancouver, BC Canada; 26 Division of Clinical and Metabolic Genetics, Department of Pediatrics, The Hospital for Sick Children, University of Toronto, 555 University Avenue, Toronto, ON M5G 1X8 Canada; 27 Division of Neurology, Department of Pediatrics, The Hospital for Sick Children, University of Toronto, Toronto, ON Canada

**Keywords:** ATP8A2, Phospholipid transfer protein, Optic atrophy, Chorea, Choreoathetosis, Dystonia, Developmental disabilities, Whole exome sequencing

## Abstract

**Background:**

*ATP8A2* mutations have recently been described in several patients with severe, early-onset hypotonia and cognitive impairment. The aim of our study was to characterize the clinical phenotype of patients with *ATP8A2* mutations.

**Methods:**

An observational study was conducted at multiple diagnostic centres. Clinical data is presented from 9 unreported and 2 previously reported patients with *ATP8A2* mutations. We compare their features with 3 additional patients that have been previously reported in the medical literature.

**Results:**

Eleven patients with biallelic *ATP8A2* mutations were identified, with a mean age of 9.4 years (range 2.5–28 years). All patients with *ATP8A2* mutations (100%) demonstrated developmental delay, severe hypotonia and movement disorders, specifically chorea or choreoathetosis (100%), dystonia (27%) and facial dyskinesia (18%). Optic atrophy was observed in 78% of patients for whom funduscopic examination was performed. Symptom onset in all (100%) was noted before 6 months of age, with 70% having symptoms noted at birth. Feeding difficulties were common (91%) although most patients were able to tolerate pureed or thickened feeds, and 3 patients required gastrostomy tube insertion. MRI of the brain was normal in 50% of the patients. A smaller proportion was noted to have mild cortical atrophy (30%), delayed myelination (20%) and/or hypoplastic optic nerves (20%). Functional studies were performed on differentiated induced pluripotent cells from one child, which confirmed a decrease in ATP8A2 expression compared to control cells.

**Conclusions:**

*ATP8A2* gene mutations have emerged as the cause of a novel neurological phenotype characterized by global developmental delays, severe hypotonia and hyperkinetic movement disorders, the latter being an important distinguishing feature. Optic atrophy is common and may only become apparent in the first few years of life, necessitating repeat ophthalmologic evaluation in older children. Early recognition of the cardinal features of this condition will facilitate diagnosis of this complex neurologic disorder.

**Electronic supplementary material:**

The online version of this article (10.1186/s13023-018-0825-3) contains supplementary material, which is available to authorized users.

## Background

P4-ATPases are a group of proteins that actively transport phospholipids across cell membranes, a process known as ‘flipping’ [1, 2]. The main structural phospholipids are distributed in a non-random manner across the lipid bilayer [3], which is essential for a range of functions including vesicle trafficking, cellular signaling and neuronal cell survival [4]. Although 14 P4-ATPases (flippases) have been identified, only two genes (*ATP8A2* and *ATP8B1*) have been associated with human disease [1, 5].

ATP8A2 is highly expressed in the brain, spinal cord, retina and testis [6, 7]. Mutations in *ATP8A2* were initially identified in a family with a clinical phenotype of cerebellar ataxia, mental retardation and disequilibrium (CAMRQ syndrome) [8]. More recently, *ATP8A2* has been linked to a phenotype of intellectual disability, severe hypotonia, chorea and optic atrophy without obvious radiographic evidence of cerebellar atrophy [6, 9, 10].

We provide a clinical summary of 9 previously unreported patients with *ATP8A2* mutations identified via whole exome sequencing. Detailed clinical information is also provided for 2 previously reported patients.^9^ We compare the clinical features of these 11 individuals with three additional published patients [6, 8, 10]. Expression studies of differentiated induced pluripotent cells from one patient revealed decreased ATP8A2 RNA expression and protein levels compared to control cells.

Our observations confirm that biallelic *ATP8A2* mutations cause a distinct clinical phenotype that is characterized by global developmental delays, severe hypotonia, optic atrophy and hyperkinetic movement disorders.

## Methods

### Patients

Eleven patients from nine families were recruited to participate in this study. The Research Ethics Board of the Hospital for Sick Children approved this study and informed consent was obtained from all families according to the Declaration of Helsinki. The family of Patient 1 consented to a skin biopsy for functional studies to be performed.

### Molecular studies

Three unrelated patients (Patients 1, 2 and 5) had exome sequencing completed at GeneDx (Gaithersburg, MD). Genomic DNA was extracted from whole blood from affected children and their parents. Exome sequencing was performed on exon targets captured using either the Agilent SureSelect Human All Exon (50 Mb) V4 kit or Clinical Research Exome kit (Agilent Technologies, Santa Clara, CA). One microgram of DNA from peripheral blood was sonicated into 300 bp fragments, which were then repaired, ligated to adaptors, and purified for subsequent PCR amplification. Amplified products were then captured by biotinylated RNA library baits in solution following the manufacturer’s instructions. Bound DNA was isolated with streptavidin-coated beads and re-amplified. The final isolated products were sequenced using either the Illumina HiSeq 2500 or 4000 sequencing system with either 2 × 100-bp or 2 × 150-bp paired-end reads (Illumina, San Diego, CA). DNA sequence was mapped to the published human genome build UCSC hg19/GRCh37 reference sequence using BWA-Mem v0.7.8 [11]. Targeted coding exons and splice junctions of known protein-coding RefSeq genes were assessed for average depth of coverage with a minimum depth of 10X required for inclusion in downstream analysis. Local realignment around insertion-deletion sites was performed using the Genome Analysis Toolkit v2.3 [12]. Variant calls were generated simultaneously on all sequenced family members using either Samtools 0.1.18 or Samtools 0.1.18 along with GATK 2.3 HaplotypeCaller [11, 12]. All coding exons and surrounding intron/exon boundaries were analyzed. CNVs were called as previously described [13]. Automated filtering removed common sequence changes (defined as > 10% frequency present in the 1000 Genomes database). The targeted coding exons and splice junctions of the known protein-coding RefSeq genes were assessed for the average depth of coverage and data quality threshold values. Whole exome sequence data for all sequenced family members was analyzed using GeneDx’s XomeAnalyzer (a variant annotation, filtering, and viewing interface for WES data), which includes nucleotide and amino acid annotations, population frequencies (NHLBI Exome Variant Server and 1000 Genomes databases), in silico prediction tools and amino acid conservation scores (Mutation Taster, PhyloP, and CADD). Variants were filtered based on inheritance patterns, gene lists of interest, phenotype, and population frequencies, as appropriate. Resources including the Human Gene Mutation Database (HGMD), 1000 Genomes database, NHLBI Exome Variant Server, OMIM, PubMed, and ClinVar were used to evaluate genes and sequence changes of interest [14–17]. Identified sequence changes of interest were confirmed in all family members by di-deoxy Sanger sequence analysis using an ABI3730 (Life Technologies, Carlsbad, CA) and standard protocols with a new DNA preparation. CNVs were confirmed in relevant family members by whole genome or exon-focused oligonucleotide array-based comparative genomic hybridization (Agilent Technologies, Santa Clara, CA), quantitative polymerase chain reaction, or multiplex ligation-dependent amplification. Patient 3 underwent exome sequencing at BGI, Denmark with subsequent annotation and interpretation at the Genome Diagnostics laboratory in Nijmegen, the Netherlands. Libraries were prepared using Agilent SureSelect Human All Exon enrichment kit version 5 (Agilent Technologies) and sequenced on an Illumina HiSeq4000 2x150bp. Reads were aligned to hg19 reference genome using BWA − 0.7.8-r455, variants were called with GenomeAnalysisTK 3.3–0-g37228af and annotated using an in-house pipeline. Exome sequencing for Patient 4 was carried out in the molecular laboratory of the Hôpital Pitié-Salpêtrière in Paris, France. Libraries were prepared from genomic DNA using Roche SeqCap MedExome kits and sequenced on an Illumina NextSeq 500 2x150bp high output (with 12 plexes). Reads were aligned to hg19 reference genome using BWA-mem, variants were called with GenomeAnalysisTK-2014.3-17-g0583018 and annotated using SNPEff-4. Custom scripts were utilized for variant filtration and prioritization. Exome sequencing for Patients 6 and 7 was carried out at Novogene using the Agilent SureSelect V6 enrichment kit with a paired-end (150 bp) protocol at a mean coverage of 50X. Reads were aligned to genome assembly hg19 with the Burrows-Wheeler Aligner (BWA) (Version 0.7.8-r455). Exome sequencing for Patients 8 and 9 was carried out at the Institute for Molecular Bioscience in Queensland, Australia. Libraries were prepared from genomic DNA using Nextera Rapid Capture Exome kits and sequenced on an Illumina HiSeq 2000 to a minimum average coverage of 95X. Reads were aligned to hg19 reference genome using BWA-mem, variants were called with GATK HaplotypeCaller v3.7 and annotated using SnpEff v4.3 m. Custom scripts were utilized for variant filtration and prioritization. Patients 10 and 11 were diagnosed at 12 de Octubre Hospital in Madrid, Spain and have been previously reported [9]. Detailed exome sequencing methods are outlined in Additional file 1: Table S1.

### Functional studies

#### Human pluripotent stem cell differentiations

Human induced pluripotent stem cells (iPSCs) were generated from control and patient fibroblasts, differentiated into endodermal lineage cells, and cell lysates were used to quantify RNA expression and concentration of ATP8A2 protein.

The human iPSCs were generated in house from BJ fibroblasts (ATCC) or patient fibroblasts by infecting with Sendai virus as outlined in the manufacturer’s protocol (Cytotune 2.0; LifeTechnologies). Cells were plated on Matrigel (Corning) after 48 h and subsequently transitioned to mTeSR-E8 media (StemCell Technologies) from days 4–7 post-infection. At 3 weeks post-infection, iPSC clones were picked into and maintained in mTeSR-E8 on Matrigel-coated 96-well plates. Clones were passaged using ReLeSR (Stem Cell Technologies) every 4–6 days until passage 15. Sendai virus transgene expression was then analyzed, and found to be absent, using Taqman (Life Technologies) and pluripotency markers assessed by immunostaining and qPCR.

hIPSCs were plated onto geltrex (1:100)-coated 12 well plates at a density of 0.5 × 10^6^ in 10/10 media [DMEM/F12, KOSR, Glutamax, P/S, 10 ng/mL Activin A (E-biosciences) and 10 ng/mL herugulin (Tocris) [18]. Differentiations began 48 h post-seeding using a modified version of Rezania et al. [19]. Briefly, cells were rinsed with 1× DPBS (Mg^2+^ and Ca^2+^ free) and then basal culture media (MCDB 131 medium, 1.5 g/l sodium bicarbonate, 1× Glutamax, 1× P/S) with 10 mM final glucose, 0.5% BSA, 100 ng/ml Activin A (E-biosciences), and 3 μM of CHIR-99021 (Sigma) was added for 1 day only. For the following two days, cells were treated with the same media without CHIR-99021 compound to generate definitive endoderm (Stage 1). On day four, cells were cultured in basal media with 0.5% BSA, 10 mM glucose, 0.25 mM ascorbic acid (Sigma) and 50 ng/ml of KGF (R&D Systems) for 2 days to generate primitive gut tube (Stage 2). To produce posterior foregut (Stage 3), cells were treated for three days with basal media with 10 mM final glucose concentration, 2% BSA, 0.25 mM ascorbic acid, 50 ng/ml of KGF, 0.25 μM SANT-1 (Tocris Biosciences), 1 μM retinoic acid (Sigma), 100 nM LDN193189 (EMD Millipore), 1:200 ITS-X (Gibco), and 200 nM TPB (EMD Millipore).

#### qPCR

Cells were lysed in Trizol and standard phenol-chloroform extraction was used to isolate RNA as previously described [20]. Following RNA extraction, Superscript III was used for reverse transcription followed by Taqman on ViiA7 384-well thermocycler. Fluorogenic probes used included FAM and the IowaBlack non-fluorescent quencher (PrimeTime; IDT). Primer/probe sequences are: 1) ATP8A2, Primer-1 TGGTTCCTACTGCCTGTTTG; Primer-2 CCTCTTTCCATTGCTATCCCG; Probe CTTGGTTTCCAGCTCCTGCACCT. 2) TBP, Primer-1 GAGAGTTCTGGGATTGTACCG; Primer-2 ATCCTCATGATTACCGCAGC; Probe TGGGATTATATTCGGCGTTTCGGGC.

#### Protein analysis and western blotting

Cells were lysed in Laemmeli buffer (2% SDS, 10% glycerol, 5% 2-mercaptoethanol, and 0.068 M Tris HCl, pH 6.8) in the absence of bromophenol blue. The solution was boiled for 10 min. The DNA was sheared by passing the sample through a 26 gauge needle followed by sonication. The sample was then centrifuged for 10 min at 1000 g and the supernatant was retained for protein concentration determination using the Bradford Protein Assay and analysis by SDS gel electrophoresis and Western blotting.

For western blotting, proteins were transferred on to Immobilon FL membranes (Millipore, Bedford, MA) using a BioRad semi-dry transfer apparatus. The blots were blocked with 1% milk in PBS for 30 min and subsequently labeled with ATP8A2 polyclonal antibody [21] and actin polyclonal (ab8227, Abcam) antibody as a loading control. The ATP8A2 and actin antibodies were diluted to a concentration of 0.3 mg/ml and 0.2 mg/ml, respectively in PBS containing 0.05% Tween 20 (PBST). The blots were incubated with primary antibodies for 1 h, washed with PBST, and subsequently incubated for 40 min. With secondary antibody (goat anti-rabbit Ig conjugated with horseradish perioxidase (Sigma) diluted 1:20,000 in PBST containing 0.1% milk) and washed with PBST prior to detection by ECL. The film was scanned on a LiCor imager and the intensity of the ATP8A2 labeled bands was quantified using Image Studio Lite software.

## Results

### Clinical features

**Fig. 1:**
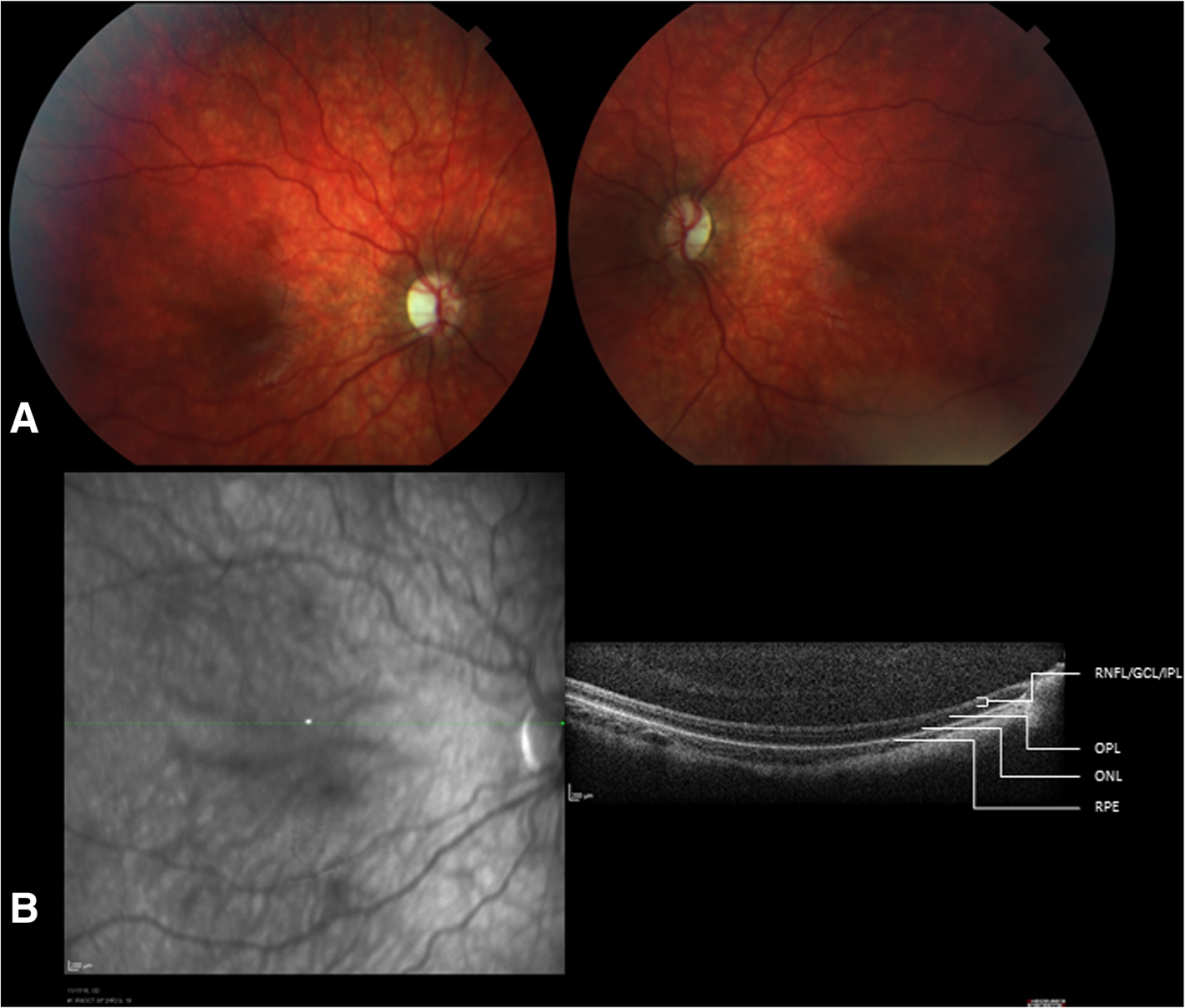
**a** Severe bilateral optic atrophy on direct funduscopic examination. **b** Optical coherence tomography (OCT) reveals relatively thinning of the inner retinal layers suggestive of optic atrophy of those neuronal elements. Outer retinal layers (OPL, ONL) are less affected. RPE = retinal pigmented epithelium; ONL = outer nuclear layer; OPL = outer plexiform layer, RNFL = retinal nerve fiber layer; GCL = ganglion cell layer; IPL inner plexiform layer


The clinical features of individuals with *ATP8A2* mutations are described for 11 patients ranging from 2.5 years to 28 years (Table 1). Patients were of varied ethnicities with 8/11 (73%) demonstrating parental consanguinity. All patients exhibited severe hypotonia at or within 6 months after birth which persisted, with older children and adults being unable to achieve head control and/or sit independently (Table 2). Expressive and receptive language skills were impaired in all patients. Most children (9/11, 82%) did not develop any meaningful speech, with only two communicating with mono or di-syllabic words, or with the aid of pictograms (Table 2). Hyperkinetic movement disorders, specifically chorea or choreoathetosis were present in all patients (Additional file 2: Video S1; Additional file 3: Video S2). Dystonia and/or facial dyskinesia were noted in a smaller proportion of patients (Additional file 4: Video S3). Feeding difficulties were a significant feature of our cohort and were reported in 10/11 (91%) individuals. All 10 patients required modified feeds such as pureed or thickened feeds, and all required significant assistance with feeding. Three patients (Patients 1, 2 and 9) required gastrostomy tubes due to their risk of aspiration when drinking thin liquids. Weight was below the third percentile for 70% (7/10) of the patients who had a recent weight available. Occipitofrontal head circumference (OFC) was within the normal range for the majority of patients, and 5/11 (45%) had OFC at or below the second percentile for age. Optic atrophy was noted in 7/9 (78%) patients who underwent funduscopic examination. The two children who did not have optic atrophy did not have testing of visual evoked potentials. In one child (Patient 1) who underwent repeat funduscopic examinations, optic atrophy was noted to progress in the first few years of life. Initial ophthalmology examination at 10 months was entirely normal including funduscopic examination, and the patient had normal visual fixation and tracking. Repeat examination at 19 months revealed mild optic nerve pallor. At 26 months she was no longer able to visually fix or follow and optic nerve atrophy was seen. Visual evoked potentials at 3 years revealed absentcortical responses. At 4.5 years she had severe optic nerve atrophy (Fig. 1) with optical coherence tomography (OCT) showing selective thinning of the inner retinal layers. A smaller proportion of patients exhibited ophthalmoplegia (45%) or ptosis (36%) (Additional file 5: Video S4). Seizures were observed in two siblings from a single family, with the remaining 9/11 patients being seizure-free. Patient 8 did not require treatment with anti-epileptics and Patient 9 had seizures which were well-controlled with valproate and carbamazepine. Neither patient had EEG studies available for review. One patient was noted to have abnormal brainstem auditory evoked potentials (BAER) responses (Patient 4) consistent with sensorineural hearing loss. This patient also had abnormal somatosensory evoked potentials (SSEP) suggesting a myelinopathy involving the dorsal column of the spinal cord. One other patient (Patient 5) was noted to have abnormal hearing.

**Table 1:** Clinical characteristics of individuals with *ATP8A2* mutations

Patients	1	2	3	4	5	6	7	8	9	10	11	Total^a^
Gender	F	F	F	F	M	F	M	M	F	F	F	
Symptom onset	Birth	Birth	Birth	Birth	6 mos	4 mos	Birth	6 mos	Birth	Birth	1 mos	
Current age (years)	5y	2.5y	2.7y	6y	5y	9y	15y	16y^b^	28y	8.5y	5.5y	
Gestational age (wk)	40	39	41	40	39	39	40	40	Term	40	39	
Birth weight (kg)	4.14	3.57	3.09	4.55	2.62	2.70	2.90	N/a	N/a	3.19	2.87	
Clinical features												
Hypotonia onset	Birth	Birth	Birth	Birth	Infancy	Infancy	Birth	Infancy	Birth	Birth	Infancy	
Hypotonia persists?	Yes	Yes	Yes	Yes	Yes	Yes	Yes	Yes	Yes	Yes	Yes	100%
Muscle weakness	Yes	Yes	Yes	N/a	No	Yes	Yes	N/a	N/a	Yes	Yes	80%; 8/10
Optic Atrophy	Yes	N/a	Yes	Yes	Yes	No	No	Yes	N/a	Yes	Yes	78%; 7/9
Ophthalmoplegia	No	No	No	Yes	No	No	No	Yes	Yes	Yes	Yes	45%
Ptosis	No	No	Yes	Yes	No	No	No	No	Yes	No	Yes	36%
Hearing Loss	No	No	No	Yes	Yes	No	No	No	No	No	No	18%
Seizures	No	No	No	No	No	No	No	Yes	Yes	No	No	18%
Feeding difficulties	Yes, G-tube	Yes, G-tube	Yes	No	Yes	Yes	Yes	Yes	Yes, G-tube	Yes	Yes	91%
Sleep disturbance	Yes	Yes	No	N/a	No	Yes	No	No	No	No	Yes	40%; 4/10
Movement disorder												
Chorea or choreoathetosis	Yes	Yes	Yes	Yes	Yes	Yes	Yes	Yes	Yes	Yes	Yes	100%
Dystonia	Yes	No	Yes	Yes	No	No	No	No	No	No	Yes	36%
Facial dyskinesia	No	No	No	No	No	No	No	No	No	Yes	Yes	18%
Current head size (OFC)	25%ile	25%ile	5%ile	< 2%ile	10%ile	2%ile	15%ile	< 3%ile	< 2%ile	25%ile	< 2%ile	
Current weight	25%ile	83%ile	60%ile	N/A	< 3%ile	< 2%ile	< 2%ile	< 3%ile	< 2%ile	< 3%ile	< 3%ile	
Current length/height	85%ile	90%ile	40%ile	N/A	25%ile	< 2%ile	< 2%ile	< 3%ile	< 2%ile	5%ile	20%ile	

^a^Denominator = 11 unless otherwise indicated; *F* female, *M* male, *y* years old, *mos* months old, *wk* weeks, *N/a* not available, *%ile* percentile, *OFC* occipitofrontal head circumference, *NCS/EMG* nerve conduction study and electromyography, *BAER* brainstem auditory evoked response, ^b^Deceased

**Table 2:** Best developmental achievement of individuals with *ATP8A2* mutations

Patient	Age (years)	Language	Gross motor	Fine motor	Feeding	G-tube
1	5	Non-verbal	Cannot sit	Transfers hand-to-hand	Requires pureed or thicker feeds	Yes
2	2.5	Babbles	Cannot sit	Holds objects, not transferring	Requires thickened foods	Yes
3	2.7	Non-verbal	Cannot roll or support head	Attempting to grasp	Requires pureed or thicker feeds	No
4	6	Non-verbal	Cannot sit	Cannot grasp	No issues	No
5	5	Non-verbal	Cannot sit	Hand grasp	Feeding difficulties	No
6	9	Non-verbal	Impaired	Impaired	Feeding difficulties	No
7	15	Non-verbal	Impaired	Impaired	Feeding difficulties	No
8	16^a^	None	Impaired	Impaired	Feeding difficulties	No
9	28	None	Impaired	Impaired	Feeding difficulties	Yes
10	8.5	Monosyllabic & disyllabic words	Cannot support head	Holds objects	Requires soft foods	No
11	5.5	Uses signs, pictograms	Cannot support head	Attempting to grasp	Requires crushed foods	No

^a^Deceased

Additional file 3:**Video S2.** Patient 7 demonstrating chorea of upper and lower limbs. (3GP 64981 kb)


Nerve conduction studies and electromyography was performed on 8/11 patients with no evidence of a sensorimotor neuropathy observed (Table 3). Single fiber EMG was normal in one patient who had ptosis. Neuroimaging was performed in all but one patient. MRI of the brain revealed no intracranial abnormalities in 50% (5/10) patients (Table 3). Non-specific findings included mild cerebral or cortical atrophy (30%), mild delay in myelination (20%), thin corpus callosum (20%) or hypoplastic optic nerves (20%). Additional detailed clinical information is provided for Patients 1 and 3 as Additional file 6.

**Table 3:** Ancillary testing of individuals with *ATP8A2* mutations

Patient	Nerve conduction	MRI brain (age of study, years)
1	Normal	Normal; hypoplastic optic nerves (2 yrs., 3 yrs)
2	N/A	Normal (1.5 yrs)
3	Normal	Mild delay in myelination for age; subcortical white matter volume loss, thin corpus callosum (8 mos, 1.3 yrs)
4	Normal	Normal
5	Normal	Normal; hypoplastic optic nerves
6	N/A	N/A
7	Normal	Normal
8	Normal	N/A (CT brain = mild cerebral atrophy)
9	Normal	Hyperintense signal (T2FLAIR) in optic radiations (12 yo)
10	Normal	Delayed myelination for age, mild cerebral atrophy, thin corpus callosum (6 yrs)
11	N/A	Delayed myelination in temporal lobes (1.8 yrs)

### Genetic testing

Biallelic *ATP8A2* gene mutations were identified in all affected individuals, consistent with autosomal recessive inheritance (Table 4). Carrier parents were asymptomatic. Identified genetic variants included missense, splicing, intragenic deletions, and frameshift mutations, which were predicted to cause loss of function of ATP8A2, and were not present in the homozygous state in the Exome Aggregation Consortium (ExAC) public database or our internal exome databases of unaffected individuals. All variants occurred within highly conserved domains, and were predicted to be damaging or possibly damaging by multiple in silico models (SIFT, Polyphen, MetaSVM, MetaSVM, Mutation Taster, PhyloP, and CADD).

**Table 4:** Genetic characteristics of individuals with *ATP8A2* mutations

Patient	Ethnicity	Alleles	Mutations	Predicted effect on protein
1	French Canadian, Algerian	Compound heterozygote	c.1185 + 5G > A del exons 28–33	Destroys spice donor site in intron 12 Partial gene deletion
2	European Ashkenazi Native American	Compound heterozygote	c.1787delA c.321 + 3_321 + 8 delAATGGT	p.Asn596MetfsX – frameshift Destroys spice donor site in intron 3
3	Turkish	Homozygous^a^	c.1756C > T	p.Arg586^*^ - premature stop codon
4	Moroccan	Homozygous^a^	c.2104 T > C	p.Trp702Arg – missense
5	Sri Lankan	Homozygous^a^	c.1286A > T	p.Lys429Met – missense
6	Iranian	Homozygous^a^	c.1474_1662del (del exons 17–18)	p.Pro492_Ala554del
7	Iranian	Homozygous^a^	c.1474_1662del (del exons 17–18)	p.Pro492_Ala554del
8	Lebanese	Homozygous	c.3188_3196delCTATGGTCC insGAAGAAG	p.Thr1063fs - frameshift
9	Lebanese	Homozygous	c.3188_3196delCTATGGTCC insGAAGAAG	p.Thr1063fs - frameshift
10	Spanish	Homozygous^a^	c.1287G > T	p.Lys429Asn - missense
11	Spanish, Argentinian	Compound heterozygote	c.1630G > C c.1873C > T	p.Ala544Pro – missense p.Arg625Trp - missense

^a^Known consanguinity

### Functional studies

**Fig. 2:**
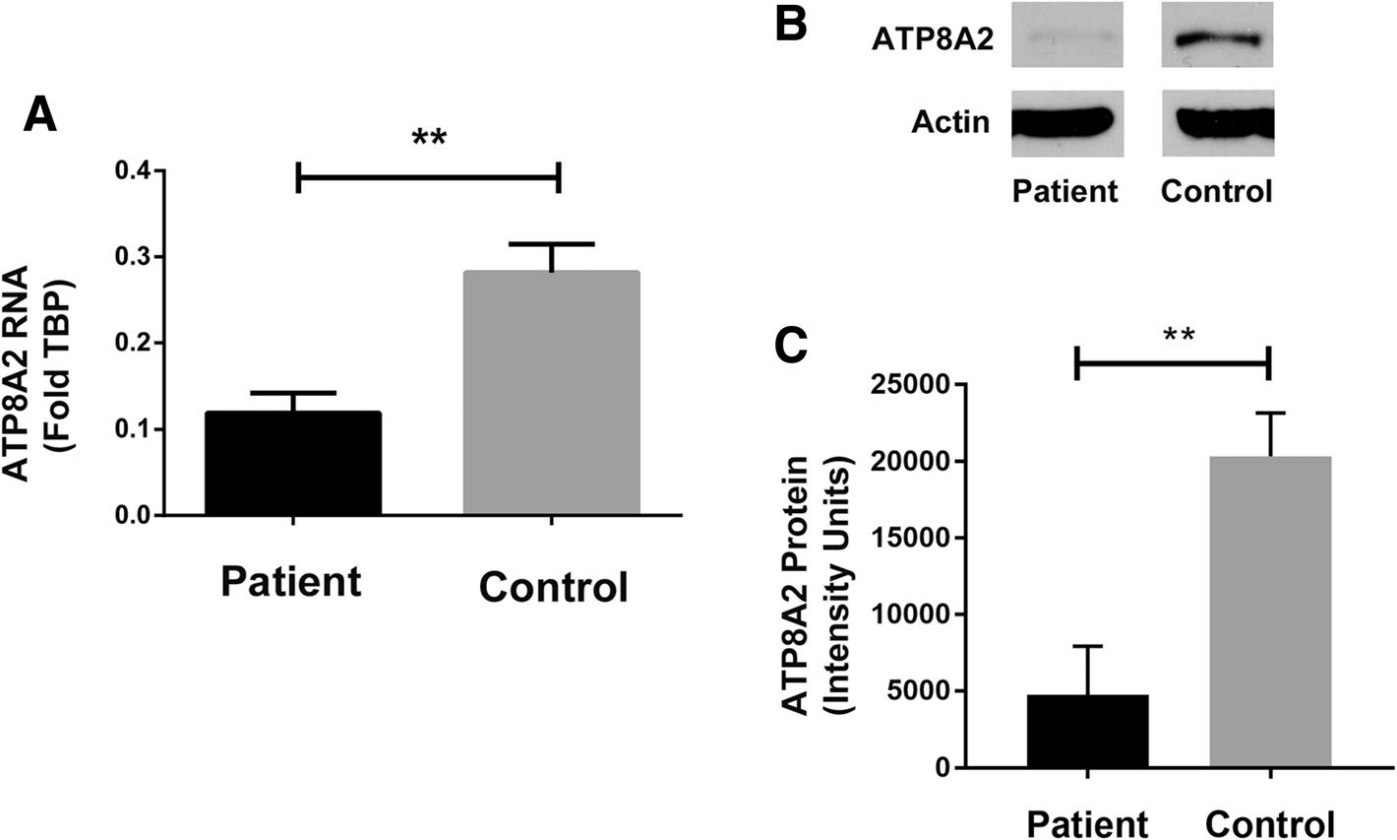
ATP8A2 expression in gut endoderm cells differentiated from patient and control fibroblasts. **a** ATP8A2 RNA expression levels were determined by Taqman qPCR; data were normalized to TBP and are expressed as an average +/− SEM for *n* = 4. **b** Representative western blots of foregut endodermal cell lysates, differentiated from control or patient induced pluripotent stem cells, and labeled for ATP8A2 and actin (loading control). **c** Quantification of ATP8A2 protein expression from western blots expressed as an average +/-SEM for n = 4. *P* values ** are ≤0.01

Initial studies were unable to detect any ATP8A2 in the control or fibroblasts from Patient 1, due to low ATP8A2 expression in this tissue. Subsequent reprogramming of the fibroblasts into induced pluripotent cells allowed derivation of tissues that expressed higher amounts of ATP8A2 to enable study. We found that early endoderm derivatives expressed high levels of ATP8A2 and allowed analyses of RNA expression and ATP8A2 protein expression. To ensure that the patient’s cells could differentiate into endoderm with equivalent efficiency as control cells, we assessed expression of two endoderm markers, FOXA2 and SOX17. Using expression of these markers, the patient’s cells more readily adopted the endoderm fate compared to control cells (Supplementary Figure S1). ATP8A2 mRNA expression was significantly lower in the patient compared to control cells and there was diminished ATP8A2 protein in the patient cells, confirming the deleterious effect of the mutations (Fig. 2). From these studies we conclude this patient had loss of function mutations in *ATP8A2*, which likely improved endoderm differentiation.

## Discussion

Despite the critical role of P4-ATPase proteins in normal cellular functioning, they have only recently been implicated in human disease. Whole exome sequencing continues to expand our clinical diagnostic capabilities, and in this instance has permitted the delineation of a specific neurologic phenotype associated with *ATP8A2* mutations.

An early report described an infant with severe hypotonia and global developmental delay who was found to have a de novo t(10;13) balanced translocation with the breakpoint disrupting the coding sequence of a single gene, *ATP8A2* [6]. Although the authors were not able to quantify ATP8A2 expression, they hypothesized that the phenotype may be attributed to haploinsufficiency of the *ATP8A2* gene since a mutation on the other allele was not identified [6]. It was not documented if this patient underwent funduscopic examination, however visual parameters at 1 year of age were reported to be normal. Repeat visual assessment was not reported to ascertain if, as for Patient 1 in our case series, optic atrophy developed later in childhood.

Four members of a consanguineous Turkish family with cerebellar ataxia, mental retardation and disequilibrium (CAMRQ) syndrome who had previously been identified by homozygosity mapping to have shared regions of homozygosity on chromosomes 13, 19 and 20 underwent whole exome sequencing [8]. A homozygous c.1128 C > G; p.I376M mutation in *ATP8A2* was identified which was predicted to change the secondary structure of the ATP8A2 protein and postulated to be the cause of the CAMRQ syndrome. Phenotypic findings that were unique to these patients included truncal ataxia with or without quadrupedal gait, dysarthric speech as well as MRI evidence of mild cerebral and cerebellar atrophy. The presence or absence of optic atrophy was not reported.

One additional child was identified by whole exome sequencing to have biallelic mutations in *ATP8A2* [10]. This child demonstrated severe axial hypotonia that was noted in the first 6 months of life. At 11 years of age the child remains non-ambulatory and is capable of speaking single-word or short sentences with marked dysarthria. MRI of the brain was normal. He was reported to have choreoathetosis, dystonia and optic atrophy documented on funduscopic examination.

*ATP8A2* is responsible for maintaining a higher concentration of phosphatidylserine at the inner surface of the phospholipid bilayer [7, 22]. Mouse models of ATP8A2 deficiency have demonstrated that *Atp8a2* mutations result in impaired axonal transport, axonal loss, failure to thrive and clinical manifestations of neurodegenerative disease which are similar to the patients we describe [7]. In addition to axonal loss in the spinal cord and retina, mice harbouring *Atp8a2* loss of function mutations have been found to have axonal degeneration affecting peripheral nerves [7]. However, none of the patients with *ATP8A2* mutations in our cohort have demonstrated any abnormalities on nerve conduction studies or electromyography (NCS/EMG) (Table 1). Patient 1 underwent a sedated NCS/EMG including concentric needle EMG of intrinsic foot muscles with no neurogenic changes identified. Due to her fluctuating ptosis, Patient 3 underwent NCS with repetitive nerve stimulation as well as EMG and stimulated single fiber EMG with no abnormalities demonstrated. The precise reason for this discrepant peripheral nervous system phenotype between mouse models of *ATP8A2* loss of function mutations and our patients is unknown, and deserves further study.

Previous work has shown that ATP8A2 is important for function of ectoderm derivatives; however, here we also demonstrate that ATP8A2 is also expressed in another germ layer early in development. In the endoderm, ATP8A2 appears to limit differentiation as patient derived induced pluripotent cells with reduced ATP8A2 expression were more readily able to adopt the endoderm fate. In the future, it will be interesting to test how ATP8A2 impacts the early embryonic development of ectodermal germ layer in order to understand when it becomes important for brain development.

## Conclusion

This is the largest report of patients with confirmed *ATP8A2* mutations to date. Despite the variability in mutation type and location, the patients in this series show remarkable phenotypic similarity with all having profound cognitive impairment, severe and persistent hypotonia and chorea or choreoathetosis. The identification of the latter feature is particularly important in distinguishing patients with *ATP8A2* mutations from the many other genetic causes of cognitive impairment. Optic atrophy and, less commonly, ophthalmoplegia and ptosis can also be seen which may be helpful in establishing this diagnosis. Feeding difficulties and failure to thrive occur frequently, and require dietary modification and occasional intervention such as G-tube placement. We suggest that this disease may be under-identified in current clinical practice, and anticipate that increased awareness and diagnosis will lead to a fuller appreciation of the clinical spectrum of *ATP8A2*-related disorders.

## Additional files

Additional file 1: **Table S1.** Exome sequencing of individuals with *ATP8A2* mutations. (XLSX 11 kb)

Additional file 2: **Video S1.** Patient 1 (age 4 years old) demonstrates chorea of her head, upper and lower extremities characteristic of patients with *ATP8A2* mutations. (MOV 7783 kb)

Additional file 4: **Video S3.** Patient 4 demonstrating dystonic posturing of her arms. (MOV 1273 kb)

Additional file 5: **Video S4.** Patient 4 demonstrating chorea as well as ptosis and ophthalmoplegia. (MOV 2274 kb)

Additional file 6: Supplementary Data - additional clinical histories. **Figure S1.** Expression of FOXA2 and SOX17 in differentiated cells from Patient 1 compared to control cells. (PDF 28 kb)

## Abbreviations

BAER: Brainstem auditory evoked response
CAMRQ: Cerebellar ataxia, mental retardation and disequilibrium syndrome
EMG: Electromyography
iPSC: Induced pluripotent stem cells
MRI: Magnetic resonance imaging
NCS: Nerve conduction studies
OCT: Optical coherence tomography
RNA: Ribonucleic acid
