# Supplementary figures and images for: Recessive mutations in ATP8A2 cause severe hypotonia, cognitive impairment, hyperkinetic movement disorders and progressive optic atrophy
Source: Orphanet J Rare Dis. 2018 May 31;13:86. doi: 10.1186/s13023-018-0825-3 (PMC6048855; doi:10.1186/s13023-018-0825-3)

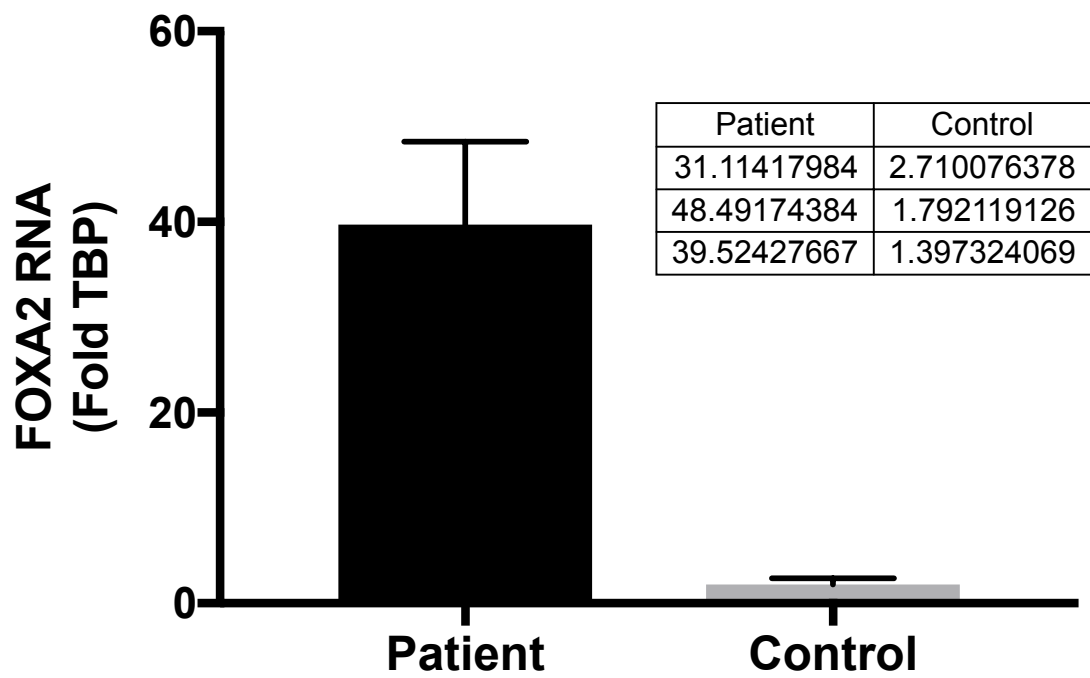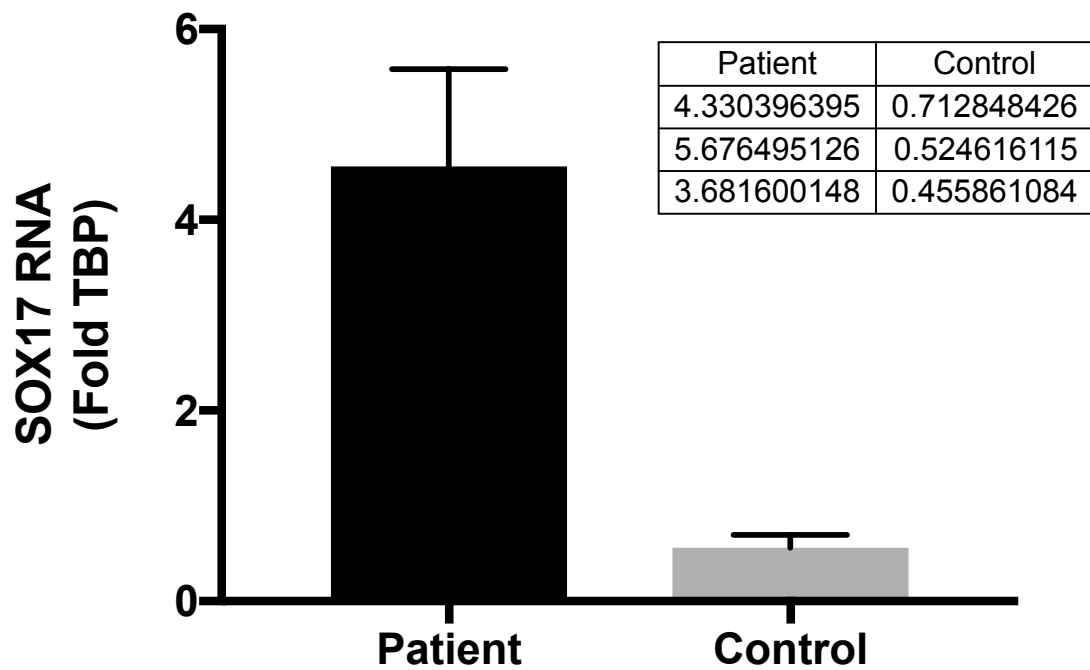

Supplement: Supplementary file 6 — Supplementary Data - additional clinical histories. Figure S1. Expression of FOXA2 and SOX17 in differentiated cells from Patient 1 compared to control cells. (PDF 28 kb) [file 13023_2018_825_MOESM6_ESM.pdf]
